# The Role of Peroxiredoxins in Cancer Development

**DOI:** 10.3390/biology12050666

**Published:** 2023-04-28

**Authors:** Pratik Thapa, Hong Jiang, Na Ding, Yanning Hao, Aziza Alshahrani, Qiou Wei

**Affiliations:** 1 Department of Toxicology and Cancer Biology, University of Kentucky College of Medicine, 1095 Veterans Dr, Lexington, KY 40508, USA; 2 Markey Cancer Center, University of Kentucky College of Medicine, 800 Rose Street, Lexington, KY 40536, USA

**Keywords:** peroxiredoxin, oxidative stress, cancer, radioresistance, chemoresistance, oxidative stress

## Abstract

**Simple Summary:**

Reactive oxygen species (ROS) are unstable derivatives of oxygen that are generated naturally as products or biproducts of biological reactions. ROS, such as hydrogen peroxide, can be utilized for defense against infectious agents and as messengers in the biological system. However, excess ROS can damage DNA, lipids, and proteins, possibly inactivating or altering their functions. The molecules utilized by the body to stop production of ROS, to neutralize excess ROS, or to repair damage caused by ROS are known as antioxidants. Peroxiredoxins (Prxs) are one of the antioxidant enzymes expressed in species from bacteria to humans that protect cells against ROS. In recent years, great progress has been made in understanding the role of Prxs in normal physiology and in different diseases. In this review, we have briefly summarized the recent findings regarding Prxs in cancer.

**Abstract:**

Peroxiredoxins (Prxs) are antioxidant enzymes with ubiquitous expression in human tissues. Prxs are expressed in archaea, bacteria, and eukaryota, often in multiple isoforms. Because of their abundant expression in different cellular organelles and extraordinary sensitivity to H_2_O_2_, Prxs are among the first defenses against oxidative stress. Prxs undergo reversible oxidation to disulfides, and some family members perform chaperone or phospholipase functions upon further oxidation. Prxs are upregulated in cancer cells. Research has suggested that Prxs can function as tumor promoters in various cancers. The major objective of this review is to summarize novel findings regarding the roles of Prxs in common cancer types. Prxs have been shown to influence differentiation of inflammatory cells and fibroblasts, remodeling of extracellular matrix, and regulation of stemness. Since aggressive cancer cells have higher intracellular levels of ROS that they can utilize to proliferate and metastasize compared to normal cells, it is critical that we understand the regulation and functions of primary antioxidants such as Prxs. These small but mighty proteins could prove to be key for improving cancer therapeutics and patient survival.

## 1. Introduction

Oxidative stress is characterized by an imbalance between oxidants and antioxidants. Reactive oxygen species (ROS), such as superoxide anion, hydroxyl radical, and hydrogen peroxide (H_2_O_2_), and reactive nitrogen species, such as nitric oxide and peroxynitrite, are oxidants produced naturally in the body by enzymatic reactions, including aerobic respiration and protein folding, or induced by external factors, such as ionizing radiation and tobacco. These reactive species are tightly regulated at the cellular level through antioxidants to act as second messengers in a variety of signaling pathways and to prevent damage to biomolecules. Antioxidants can be enzymatic (for example: superoxide dismutase, catalase, and peroxiredoxin) or non-enzymatic (for example: Glutathione (GSH) and vitamin E). The study of antioxidants is important since oxidative stress has been linked to cardiovascular diseases, neurodegenerative diseases, and cancer.

Peroxiredoxins (Prxs) are a family of thiol proteins that catalyze reduction of H_2_O_2_, alkyl hydroperoxides, and peroxynitrite to water, corresponding alcohols, and nitrite, respectively, using thioredoxin (Trx) or GSH as reductants [1,2,3]. In addition to peroxidase function, Prxs can also function as chaperones and regulators of the circadian clock [4,5]. Prx was first identified in yeast in 1987 and in *S. typhimurium* in 1990 [6,7]. In the following years, it was discovered that Prxs are ubiquitously expressed in all living organisms, and the name peroxiredoxin was first suggested in 1994 [8,9]. All Prxs contain a highly reactive cysteine called ‘peroxidatic’ cysteine (C_P_) that is oxidized by peroxides to form sulfenic acid (C_P_SOH). Some Prxs contain a second reactive cysteine called ‘resolving’ cysteine (C_R_) that forms a disulfide bond with C_P_SOH. In mammalian cells, six isoforms of Prxs have been discovered: Prx1–6. They can be classified into three subfamilies based on the location of C_R_:

1. Typical 2-Cys Prx. Prx1 to Prx4 are typical 2-Cys Prxs. A catalytic unit consists of a homodimer where both subunits contain C_P_ and C_R_. Oxidized C_P_ (for example, C_P_SOH) of one subunit forms disulfide with C_R_SH of another subunit. This disulfide bond is typically reduced by thioredoxin reductase (TrxR) or GSH–glutaredoxin (Grx) reductase systems. Typical 2-Cys Prxs exist in dimers and decamers (dodecamers for Prx3), with the ratio influenced by oxidation of C_P_. These Prxs can also function as protein chaperones.
2. Atypical 2-Cys Prx. Prx5 is considered atypical 2-Cys Prx. Oxidized C_P_ of a Prx5 molecule forms disulfide with C_R_SH in the same molecule. This disulfide bond is also reduced by the Trx–TrxR system. Unlike typical 2-Cys Prxs, Prx5 does not form decamers.
3. 1-Cys Prx. Prx6 does not contain C_R_; therefore, disulfide bond formation takes place with other thiol proteins, such as π glutathione S-transferase (πGST), and is reduced by GSH [10]. Unlike other Prxs, Prx6 expresses phospholipase A_2_ (PLA_2_) activity.

A different classification system exists based on profiling of active site structure and sequence information: (1) Prx1, (2) Prx5, (3) Prx6, (4) Tpx, (5) PrxQ, and (6) AhpE [11]. Mammalian Prx1–Prx4 belong to the Prx1 subfamily, while mammalian Prx5 and Prx6 are classified under Prx5 and Prx6 subfamilies, respectively. The other subfamilies are not expressed in mammalian cells.

## 2. Structural Aspects and Subcellular Distribution

Human Prxs have nucleotide and amino acid sequences that are similar to commonly used animal models, such as mice and zebrafish (Table 1) [12]. Prx protein core comprises seven β-strands and five α-helices [13]. Prxs also contain a Trx-like fold that is essential for peroxidatic function. Trx fold is composed of a central core of five β-sheets that are surrounded by four α-helices [14]. Multiple variations of Trx fold are observed in proteins such as arsenate reductase, protein disulfide isomerase, and Prxs. Prxs contain an N-terminal extension and an insertion between the α2 and β2 of the Trx fold [15]. C_P_ is found on the N-terminus, surrounded by three highly conserved residues (proline, threonine, and arginine), leading to stabilization of this cysteine and pKa of 5–6 [16]. A conformational change occurs when C_P_ is oxidized so it can form a disulfide bond with C_R_ of another subunit, or in the case of Prx6, with another thiol protein. In Prx1–Prx4, C_R_ is located 121 amino acid residues away from C_P_ on a conserved region of the Trx-like fold. In Prx5, C_R_ is 104 residues away from C_P_ on a less conserved region of the Trx-like fold [17].

**Figure 1:**
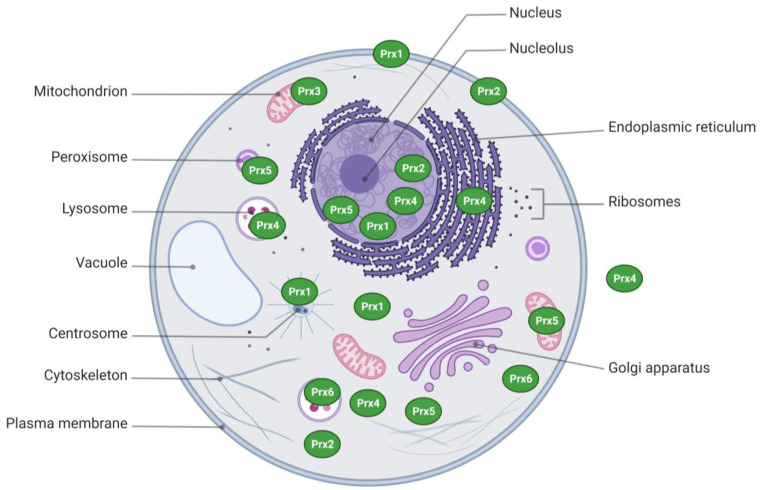
Subcellular localization of Prxs. Single subunits are shown for simplicity.

Human Prx1 is located on chromosome 1 (1p34.1), and it is transcribed from seven exons. Prx1 is distributed mainly in the cytosol, but it has also been detected in the nucleus, plasma membrane, and centrosome (Figure 1) [18]. In addition to overoxidation, Prx1 activity is also inhibited by phosphorylation. Prx1 at the plasma membrane was phosphorylated at Tyr194 by a Src kinase, resulting in decreased peroxidase activity [19]. Prx1 in the centrosome can also be inactivated by phosphorylation at Thr90 by cyclin-dependent kinases [20].

Human Prx2 is located on chromosome 19 (19p13.13) and consists of six exons. Prx2 is distributed mainly in the cytosol, and it has also been detected in the nucleus and plasma membrane [18,21]. Prx2 is reported to be more sensitive to hyperoxidation than Prx1 and Prx3 [22,23].

Human Prx3 is located in chromosome 10 (10q26.11) and consists of seven exons. Prx3 is found in the mitochondria, where it is estimated to neutralize over 90% of mitochondrial hydrogen peroxide [24].

Human Prx4 is found on chromosome X (Xp22.11). Two variants of Prx4 are expressed: somatic Prx4, which contains conventional exon 1 and exons 2–7, and testis-specific Prx4, which contains alternative exon 1 along with exons 2–7. Prx4 is distributed mainly in the endoplasmic reticulum, but it has also been detected in the extracellular matrix, cytosol, and lysosome [25,26]. We have recently summarized the roles of Prx4 in inflammation and cancer [27].

Human Prx5 is found on chromosome 11 (11q13.1) and consists of six exons. In the cells, it is distributed widely in cytosol, mitochondria, peroxisome, and nucleus [17,28]. Unlike other Prxs, which form antiparallel homodimers, Prx5 forms nonantiparallel homodimers [29].

Human Prx6 is located on chromosome 1 (1q25.1) and is transcribed from five exons. Prx6 is distributed in cytosol and lysosome [10,30]. Prx6 has PLA_2_ activity in addition to peroxidase activity. PLA_2_ function is active in acidic conditions and is increased by phosphorylation at Thr177 [31]. Thr177 phosphorylation does not affect the peroxidase activity of Prx6.

## 3. Peroxidase Mechanism

In all six isoforms of Prxs, the C_P_SH is oxidized to sulfenic acid (C_P_-SOH) by peroxides and peroxynitrite (substrate shown as H_2_O_2_ in reactions below for simplicity). In 2-Cys Prxs, the resulting sulfenic acid forms a disulfide bond with C_R_-SH of another subunit, which is then reduced, preferably by Trx [32,33].

$$Prx\left(CP-SH\right)+H_{2}O_{2}\overset{}{\to }Prx\left(CP-SOH\right)+H_{2}O$$

$$Prx\left(CP-SOH\right)+Prx\left(CR-SH\right)\overset{}{\to }Prx\left(CP-S-S-CR\right)Prx+H_{2}O$$

$$Prx\left(CP-S-S-CR\right)Prx+(SH-)Trx(-SH)\overset{}{\to }Prx\left(CP-SH\right)+Prx\left(CR-SH\right)+Trx(S-S)$$

In Prx5, the C_P_-SOH forms an intrasubunit disulfide with C_R_-SH before reduction by Trx [29].

$$Prx\left(CP-SOH\right)\overset{}{\to }Prx\left(CP-S-S-CR\right)+H_{2}O$$

$$Prx\left(CP-S-S-CR\right)+\left(SH-\right)Trx\left(-SH\right)\overset{}{\to }Prx\left(CP-SH\right)+Trx(S-S)$$

In Prx6, the C_P_-SOH forms a heterodimeric disulfide with SH of πGST before reduction by GSH [10].

$$Prx\left(CP-SOH\right)+\pi GST\left(C-SH\right)\overset{}{\to }Prx\left(CP-S-S-C\right)\pi GST+H_{2}O$$

$$Prx\left(CP-S-S-C\right)\pi GST+GSH\overset{}{\to }Prx\left(CP-S-S\right)G+\pi GST(C-SH)$$

$$Prx\left(CP-S-S\right)G+GSH\overset{}{\to }Prx\left(CP-SH\right)+GSSG$$

In the presence of high concentrations of peroxides, typical 2-Cys Prxs can become hyperoxidized and overoxidized instead of undergoing disulfide formation. Hyperoxidized 2-Cys Prxs can be reduced by the enzyme sulfiredoxin (Srx) in an ATP-dependent manner [34]. Mammalian Prx5 and Prx6 are more resistant to hyperoxidation, and they cannot be reduced by Srx [35].

$$Prx\left(CP-SOH\right)+H_{2}O_{2}\overset{}{\to }Prx\left(CP-SOOH\right)+H_{2}O$$

$$Prx\left(CP-SOOH\right)+ATP\overset{Srx}{\to }Prx\left(CP-SOH\right)+ADP$$

$$Prx\left(CP-SOOH\right)+H_{2}O_{2}\overset{}{\to }Prx\left(CP-SOOOH\right)+H_{2}O$$

## 4. Prxs and Cancer

Prxs are frequently dysregulated in cancer and are being increasingly associated with cancer initiation and metastasis. Experimental data using in vitro and in vivo models have shown the redox-dependent and independent roles of Prxs in oncology. Below, we have summarized recent progress in Prx1–Prx6 in major cancers.

### 4.1. Prxs Promote Carcinogenesis

Prx1 is positively associated with colitis and colon cancer. Two-dimensional agarose gel electrophoresis (2-DE) followed by mass spectrometry analysis of proteins isolated from biopsies (sigmoid colon) in two patients with active ulcerative colitis (UC), two patients with inactive ulcerative colitis, and four healthy subjects showed that Prx1 is upregulated in active UC compared to inactive UC and healthy controls [36]. Oxidized Prx1 protein levels were higher in healthy and inactive UC groups while reduced Prx1 level was higher in the active UC group. Immunohistochemical (IHC) staining of patient samples confirmed that Prx1 increased with increasing inflammation in mucosal crypts [36]. Furthermore, IHC staining of Prx1 in 22 normal mucosae, 6 UC-associated low-grade dysplasias, 5 high-grade dysplasias, and 5 UC-associated carcinomas detected increasing Prx1 expression in dysplasia and carcinoma [36]. Further studies are warranted to establish a causal relationship between Prx1 and colon tumorigenesis in inflammation-associated sporadic colorectal cancer (CRC) as well as hereditary CRC models.

In breast cancer, loss of Prx1 due to reduced zinc (Zn) intake is linked to tumor formation [37]. Bostanci et al. treated offspring of nulliparous mice fed control (ZA, 30 mg Zn/kg) or a marginal Zn diet (ZD, 15 mg Zn/kg) with corn oil or 7,12-dimethylbenz(a)anthracene (DMBA, 1 mg/wk) for 4 weeks. Mice fed ZD had shorter tumor latency and greater incidence of non-palpable tumors. Mechanistic studies showed reduced protein levels of Prx1 and p53 and higher oxidative DNA damage in mammary tissue of mice fed ZD. The authors propose that Zn deficiency compromises the antioxidant capacity of mammary cells, leading to higher oxidative stress and carcinogenesis [37]. This points to the need to delineate the role of diet components, such as Zn, in transcriptional and translational regulation of Prxs in normal physiology and cancers. In addition, Prx1 inhibits the cancer-associated fibroblast-like phenotype in breast cancer [38]. Primary mammary fibroblasts (MFs) isolated from Prx1 knockout mice had increased α-SMA, collagen, and Vimentin compared to Prx1 wildtype MFs. Mechanistic studies revealed that Prx1 knockdown MFs had increased oxidation of PTEN and phosphorylation of JNK when treated with H_2_O_2_. JNK1 binds to reduced Prx1 but not to overoxidized Prx1. Thus, Prx1 prevents corrupt activation of MFs [38].

Prx2 enhances intestinal tumorigenesis induced by *APC* mutation [39]. Prx2 homozygous knockout mice developed significantly fewer small intestine and colon tumors and had longer survival compared to Prx2 heterozygous and Prx2 wildtype groups in an APC^Min/+^ mouse model. Prx2 knockdown increased H_2_O_2_ accumulation and decreased total β-catenin protein levels in APC-mutant HT-29 and SW480 cells. β-catenin reduction could be blocked by inhibition of proteasomes and GSK3β. Immunoprecipitation (IP) assay revealed that Prx2 increased Axin1 complexes by blocking PARylation/ubiquitination of Axin1 [39]. In an in vitro PARP assay, the authors found that Prx2 loss impaired tankyrase activity in HT-29 and SW480 cell lines. In summary, Prx2 promotes intestinal tumorigenesis by inhibiting β-catenin degradation.

In a urethane-induced lung cancer model, human Prx4-expressing transgenic mice developed larger tumors than non-transgenic control mice [40]. IHC staining of extracted tumors showed increased cell proliferation, decreased oxidative DNA damage and apoptosis, and increased microvascular permeability and macrophage infiltration in Prx4 overexpressing tumors. Western blot analysis of tumor tissues showed increased p-c-Jun and p-p65 in Prx4-overexpression tumors, suggesting the involvement of NF-κB and AP-1 pathways [40]. Similarly, we have recently shown that Prx4 knockout FVB/N mice developed a reduced number and size of tumors compared to wildtype FVB/N mice in azoxymethane/dextran sulfate sodium (AOM/DSS)-induced colorectal cancer and urethane-induced non-small cell lung cancer (NSCLC) [41,42]. Loss of Prx4 reduced tumor cell proliferation in the lung cancer model and increased tumor cell death in the colorectal cancer model. Our studies also report novel functions of Prx4 in promoting immune infiltration into the tumors as well as regulating cytokine secretion from the immune cells [41,42]. Thus, Prx4 promotes tumor formation in lung cancer and colorectal cancer.

Prx6 also promotes lung tumorigenesis in animal models [43]. Presenilin 2 (PS2) N141I transgenic mice developed significantly lower spontaneous lung cancer compared to wildtype transgenic mice. The authors found that mutant PS2 transgenic mice tumors had over 500 times lower Prx6 expression compared to wildtype [43]. Accordingly, both peroxidase and phospholipase activities were lowered in mutant PS2 transgenic mice compared to their wildtype transgenic counterpart. IHC staining of Prx6 in human lung cancer tissue array showed overexpression of Prx6 in tumors compared to normal tissues. In addition, the authors discovered a 50% increase in PLA_2_ activity in cancer tissues compared to normal tissues. In IP assay, Prx6 and PS2 co-localization was increased in PS2 mutant skin fibroblasts AG09908 cells compared to non-mutated epithelial cells A431 cells [43]. Immunofluorescence analysis proved that this co-localization could be reversed by treatment of γ-secretase inhibitor L685,458. Prx6 and PS2 co-localization was increased in urethane-induced lung tumors isolated from mutant PS2 transgenic mice compared to wildtype. IP analysis of A549 and NCIH460 also demonstrated that, compared to wildtype PS2, the mutant PS2 had a higher affinity for Prx6 [43]. Transfection of mutant PS2 plasmid into A549 and H460 cells inhibited Prx6 expression and cell viability and increased PLA_2_ cleavage and γ-secretase activity compared to wildtype PS2 transfection or vector transfection. Thus, PS2 mutation inhibits the PLA_2_ activity of Prx6 to suppress lung tumor development [43].

### 4.2. Prxs Regulate Cancer Progression

#### 4.2.1. Prx1

In lung cancer, Prx1 protects cells against apoptosis and promotes invasion in vitro. Knockdown of Prx1 in A549 cells upregulated E-cadherin at the protein level and suppressed TGF-β-induced cell migration [44]. Using a luciferase activity assay, it was shown that catalytic Cys51 of Prx1 was critical in regulation of E-cadherin expression. The mechanism of how Prx1 peroxidase activity is used to regulate E-cadherin is not understood. In a different study, Prx1 overexpression increased anchorage-dependent colony formation and Matrigel invasion of A549 cells [45]. In A549 cells, inhibition of Prx1 with the small molecule AMRI-59 caused apoptosis [46]. AMRI-59 treatment activated both mitochondria- and apoptosis signal-regulated kinase-1-mediated signaling pathways, resulting in cell death. This could be prevented by Prx1 overexpression or N-acetyl cysteine (NAC) pretreatment. AMRI-59 was later discovered to act as a radiosensitizer in non-small cell lung cancer cells [47]. In a clonogenic assay of H460 and H1299, pretreatment with AMRI-59 increased sensitivity of these cells to irradiation. Western blot analysis showed an increase in cleaved caspase 3 upon combined treatment with ionizing radiation (IR) and AMRI-59, and cell survivability could be rescued by pan-caspase inhibitor z-Vad-Fmk. Similarly, combined treatment with IR and AMRI-59 induced ROS production (measured using DCFDA assay) and oxidative DNA damage (measured using γH2AX immunofluorescence staining), both of which could be rescued by NAC [47]. Subcutaneous injection of these NSCLC cell lines in BALB/c nu mice followed by various modes of treatments showed the combination of IR and AMRI-59 to be the most effective approach. Western blot analysis of cells in vitro showed that the combined effect of IR and AMRI-59 could be further increased by CREB-1 inhibitor [47]. Thus, inhibition of Prx1 is a novel approach to overcome radioresistance in NSCLC.

Prx1 protects hepatoma cells against apoptosis in vitro [48]. Knockdown of Prx1 in the HCC cell line decreased cell proliferation and increased apoptosis. This was associated with upregulation of Bax protein level and activation of mitochondrial fission as indicated by elevated Drp1, Fis1, and Dyn2 protein levels. Prx1 also contributes to epithelial–mesenchymal transition (EMT) in head and neck squamous cell carcinoma (HNSCC): Long non-coding RNA LINC00460, which enhances HNSCC cell proliferation and metastasis, physically interacts with Prx1 and facilitates Prx1 entry into the nucleus [49]. Prx1, in turn promotes the transcription of LINC00460, forming a positive feedback loop. In addition, overexpression of Prx1 upregulated Zeb1, Zeb2, Vimentin, and N-cadherin at mRNA and protein levels. Using quantitative real-time polymerase chain reaction (qRT-PCR) of paired HNSCC and adjacent normal tissues, the authors also demonstrated that high levels of LINC00460 and Prx1 expression were positively associated with lymph node metastasis and tumor size in HNSCC patients [49].

Several studies have shown that Prx1 promotes prostate cancer survival and migration. Prx1 was identified to interact with tumor protein D52 (TDP52) in the LNCaP cell line via GST pull down assay and 2-D mass spectrometry [50]. Increasing TDP52 induction by doxycycline in LNCaP and PC3 cells caused an increase in Prx1 levels, suggesting that TDP52 causes dimerization of Prx1. When these cell lines stably expressing TPD52 were exposed to increasing concentrations of H_2_O_2_ (from 0 μM to 100 μM), tandem affinity purification of TPD52 showed an increase in the fraction of Prx1 purified. This indicates that Prx1 interaction with TPD52 increases with oxidative stress. Knockdown of either Prx1 or TDP52 caused comparable reduction in cell proliferation and cell migration of LNCaP and PC3 cells [50]. Prx1 also promotes prostate cancer growth by activating androgen receptor (AR) signaling [51]. Overexpression of TXNDC9, which can also be induced by tunicamycin, increased AR protein levels in LNCaP, VCaP, and C4-2B cell lines. GST pull down assay followed by mass spectrometry identified Prx1 and MDM2 as two of the major interacting proteins of TXNDC9 in LNCaP and VCaP cell lines. AR protein levels in the presence of tunicamycin were reduced by knockdown of Prx1 but were enhanced by overexpression of Prx1. In addition, knockdown of TXNDC9 reduced the expression of prostate-specific antigen (PSA) in LNCaP cells, and this could be reversed by overexpression of Prx1 under tunicamycin treatment [51]. Transfection of increasing amounts of Flag-Prx1 plasmid into LNCaP and C4-2B cells followed by Western blot analysis showed that the binding between MDM2 and AR decreased steadily in the presence of tunicamycin. The authors suggest that Prx1 competing with MDM2 to bind with AR may facilitate MDM2-mediated degradation of TXNDC9. The combination of ConoidinA (which inhibits Prx1) and Enzalutamide (which inhibits AR) reduced the cell viability of C4-2B cells more significantly when compared with single treatments [51]. Thus, combined inhibition of Prx1 and AR or disruption of Prx1–TDP52 interaction might represent a promising treatment strategy for prostate cancer treatment.

Prx1 also promotes colorectal cancer progression. IHC staining of Prx1 in 60 colorectal cancer patient tissues showed positive Prx1 expression in 70% of the samples [52]. Prx1 expression was associated with microvascular density (measured using CD34 staining), tumor grade, metastasis, and shorter survival of patients. Wound healing and transwell Matrigel invasion assays demonstrated that Prx1 knockdown in HCT116 decreased migration and invasion, while Prx1 overexpression in HT-29 increased these phenotypes in vitro [52]. Three-dimensional co-culture of human umbilical vein endothelial cells (HUVEC) with CRC cell lines showed that tube formation decreased in Prx1-depleted cell lines and increased in Prx1 overexpression cell lines. Finally, MMP2, MMP9, and VEGF were downregulated in Prx1 knockdown cells and upregulated in overexpression cells as detected by Western blot analysis, further suggesting that Prx1 promotes CRC angiogenesis and metastasis [52]. Qu et al. studied the role of potential tumor suppressor miR-431-5p in colorectal cancer [53]. CRC tissues and cell lines had lower miR-431-5p expression than adjacent normal tissues and normal epithelial cell lines. Prx1 was identified as a potential target through bioinformatics analysis and confirmed in vitro. After co-culture, human umbilical cord mesenchymal stem cell (hUCMSC)-derived exosomes inhibited miR-431-5p increased cell proliferation, migration, and invasion in LoVo cells compared with negative control–-inhibitor–-exosome treatment. This effect was not observed in Prx1 knockdown cells, further confirming that miR-431-5p exerts tumor suppressive functions through Prx1 [53]. Prx1 also promotes degradation of pro-apoptotic protein NOXA to increase CRC survival [54]. Western blot analysis of apoptosis regulators showed a significant increase of NOXA in HCT116 Prx1 knockdown cells and a decrease of NOXA in SW480 Prx1 overexpression cells. In cycloheximide assay, NOXA had longer half-life in shPrx1 cells. MG132 treatment followed by co-immunoprecipitation revealed lower ubiquitination of NOXA in shPrx1 cells, suggesting that Prx1 promotes ubiquitin-mediated degradation of NOXA. Depletion of Prx1 in HCT116 reduced the neddylation of CUL5 (which activates CRL5 to ubiquitinate NOXA). In anti-CUL5 immunoprecipitation, knockdown of Prx1 reduced the amount of UBE2F, suggesting Prx1 enables their interaction [54]. Knockdown of Prx1 sensitized HCT116 cells to apoptosis induced by etoposide, and overexpression of Prx1 increased resistance of SW480 cells against etoposide. Time-dependent increase in CUL5 neddylation was observed in HCT116 upon etoposide treatment, but this effect was not seen in Prx1 knockdown cells. This indicates that cancer cells utilize Prx1-mediated CUL5 neddylation to survive against chemotherapeutics [54]. Thus, Prx1 supports angiogenesis and survival of CRC cells.

Prx1 has been reported to promote breast cancer proliferation and survival through its peroxidase function. In breast cancer cell lines MCF-7 and ZR-75-1, depletion of Prx1 decreased cell proliferation and anchorage-dependent colony formation [55]. Implantation of control and Prx1 knockout MCF-7 cells into the mammary fat pad of nude mice resulted in significantly slower tumor growth rate in the knockout group. As expected, Prx1-depleted cells were more sensitive to glucose oxidase-induced cell death. Prx2-SO_3_ and Prx4-SO_3_ levels were significantly higher in Prx1 knockout cells compared to control after glucose oxidase treatment, suggesting that Prx1 protects these 2-Cys Prxs from oxidation in breast cancer. Interestingly, glucose oxidase and another prooxidant agent, sodium L-ascorbate, also reduced the viability of breast cancer cell lines T47D, MDA-MB-231, HCC 1806, and SK-BR-3, but not of non-malignant cell line MCF-10A [55]. Other studies have also shown that Prx1 protects breast cancer cells against oxidative stress-induced cell death. Prx1 depletion increased sensitivity of triple-negative breast cancer cell lines MDA-MD-231 and HCC1806 to ascorbate and menadione [56]. Deacetylation and inhibition of Prx1 by SIRT2, a protein deacetylase, sensitized breast cancer cells to prooxidants menadione and arsenic trioxide [57]. Finally, loss of Prx1 also increases susceptibility of breast cancer cells to radiation [58]. Skoko et al. reported that Prx1 binds to and protects Rad51 cysteines from oxidation. Consequently, Prx1 knockdown sensitized MDA-MD-231 cells to irradiation-induced cell death by preventing Rad51-mediated homologous recombination DNA repair.

**Figure 2:**
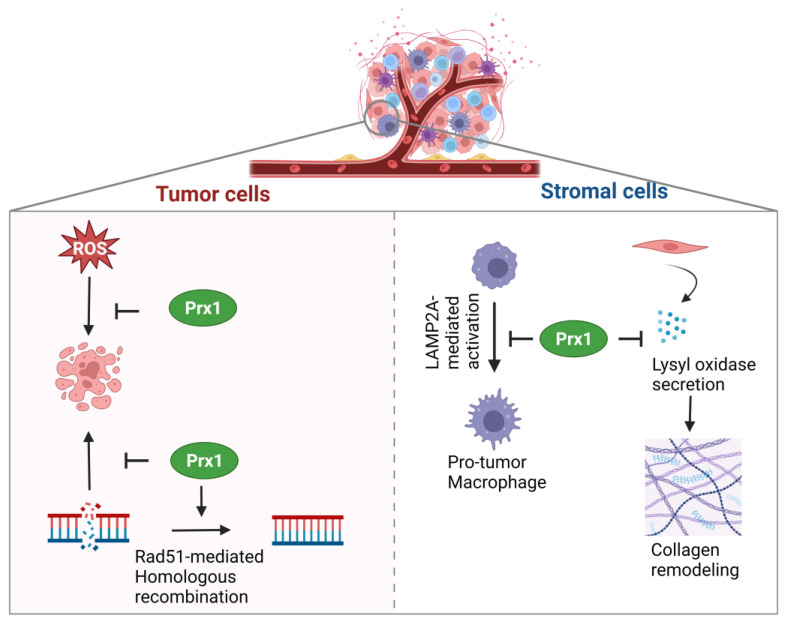
Contradictory roles of Prx1 in breast cancer tumor microenvironment. Prx1 expression in tumor cells facilitates cell survival. Prx1 prevents pro-tumorigenic activation of macrophages and collagen remodeling to counter tumor progression.

While Prx1 supports growth and survival of breast cancer cells, Prx1 expression in stromal cells of the breast tumor microenvironment is associated with inhibition of cancer progression. Loss of Prx1 prompts collagen remodeling known to promote breast cancer development [59]. Knockdown of Prx1 in mammary fibroblasts followed by injection into mammary fat of BALB/c mice resulted in an enrichment of intratumoral collagen in the shPrx1 group. In vitro studies indicated that Prx1-depleted mammary fibroblasts had higher α collagen, β collagen, and β/α collagen ratio [59]. Conditioned media derived from MDA-MB-231 cells caused Y194 phosphorylation of Prx1 (known to inactivate peroxidase activity), and this could be reversed by co-treatment with Src inhibitor PP1. shPrx1 mammary fibroblasts had increased secretion of lysyl oxidase (LOX). Per IP assay of LOX in HEK293T cells, endogenous Prx1 interacts with LOX, but this interaction was decreased when Prx1 was phosphorylated, resulting in increased extracellular LOX accumulation and collagen remodeling (Figure 2) [59]. Another study suggested that Prx1 mediates tumor suppressor activity of cytoskeletal protein transgelin-2 (TAGLN2) [60]. Knockdown of TAGLN2 in MDA-MB-231 increased cell migration in vitro and increased lung metastasis in a tail-vein injection mouse model. IP of TAGLN2 in MDA-MB-231 lysates followed by mass spectrometry analysis showed that TAGLN2 binds to Prx1. Knockdown of TAGLN2 in MDA-MB-231 caused downregulation of the Prx1 protein. Accordingly, DCFDA assay demonstrated that TAGLN2 knockdown resulted in higher ROS production [60]. Thus, interaction of Prx1 with LOX and TAGLN2 plays anti-tumorigenic role. The direct or indirect mechanism of how TAGLN2 upregulates Prx1 needs to be examined. Furthermore, the importance of peroxidase function in Prx1 in its interaction with TAGLN2 remains to be seen.

Prx1 also inhibits pro-tumorigenic activation of macrophages in breast cancer [61]. Wang and Liu et al. discovered that lysosome-associated membrane protein type 2a (LAMP2a) is upregulated in tumor-associated macrophages (TAMs) by tumor cells. Depletion of LAMP2a in macrophages reduced tumor growth in vitro and in vivo. IP of LAMP2a in bone marrow-derived macrophages treated with tumor supernatant revealed that LAMP2a binds to Prx1 [61]. Knockdown of Prx1 reversed the effects of LAMP2a knockdown in mouse hematopoietic stem cells in vitro. The authors suggest increased oxidative stress (measured using H_2_O_2_ accumulation) caused by knockdown of LAMP2a in bone marrow-derived macrophages are likely to cause pro-inflammatory activation in macrophages [61]. Finally, Prx1 protects natural killer cells from oxidative stress in the breast cancer microenvironment [62]. The authors performed bioinformatics analysis of non-stimulated human primary T cells, B cells, and Natural Killer (NK) cells and found that Prx1 transcript was significantly lower in NK cells. This was confirmed by qRT-PCR and Western blot analysis in vitro. Priming NK cells with cytokine IL-15 protected cells from glucose oxidase-induced cytotoxicity via upregulation of Prx1. Stable overexpression of Prx1 in primary NK cells and NK-92 cell line further improved cell viability under oxidative stress. Intra-tumoral transplantation of PD-L1-CAR NK cells overexpressing Prx1 in NSG mice showed increased survival and proliferation of Prx1 overexpression cells compared to control cells [62]. Thus, overexpression of Prx1 might be a useful approach to improve CAR NK-based immunotherapy in breast cancer. 

#### 4.2.2. Prx2

Prx2 promotes progression of non-small cell lung cancer (NSCLC). Western blot analysis showed that the expression of Prx2 in NSCLC cell lines is higher than in normal bronchial epithelial cells (BEAS-2b) [63]. Knockdown of Prx2 in A549 cells reduced cell proliferation, migration, and invasion. Subcutaneous injection of Prx2 knockdown A549 cells resulted in slower tumor growth compared to control cells. IHC staining of extracted tumors revealed a decrease in cell proliferation in the shPrx2 group. Tail-vein injection of A549 cells resulted in fewer metastatic nodules in Prx2 knockdown group compared to control, and this was associated with higher E-cadherin and lower Vimentin and Slug expression [63]. Jing et al. have reported similar findings in A549 and H1299 cell lines. They also discovered that loss of Prx2 reduced the phosphorylation of AKT and mTOR [64]. In addition, Prx2 promoted the stemness of drug-resistant cancer stem cells. Knockdown of Prx2 reduced colony formation and sphere formation, increased ROS (DCFDA assay) and apoptosis, and reduced migration and invasion of gefitinib-resistant A549 (A549/GR) CD133^+^ cells [65]. The authors validated that microRNA miR-122 targets Prx2 and showed that overexpression of miR-122 also suppressed proliferation, migration, and invasion of A549/GR CD133^+^ cells. In mechanistic studies, the authors used Western blot analysis to show that miR-122-mediated downregulation of Prx2 resulted in reduced activation of the Hedgehog, Notch, and Wnt/β-Catenin signaling pathways in A549 cells [65]. Finally, loss of Prx2 activity resulted in death of lung cancer cells. S-nitrosoglutathione (GSNO) nitrosylates Prx2 on Cys51 and Cys172, resulting in H_2_O_2_ accumulation and apoptosis in A549 and NCI-H1299 cells [66]. GSNO-induced H_2_O_2_ increased phosphorylation of AMPK and inhibited deacetylation activity of SIRT1, leading to cell death. Thus, Prx2 aids survival and malignancy of NSCLC through a variety of pathways.

Prx2 increases growth and progression of CRC. Knockdown of Prx2 using shRNAs reduced proliferation of HCT116 and LoVo cell lines [67]. Flow cytometry analysis proved that Prx2 knockdown caused increased cell cycle arrest in G2/M phase in HCT116 and G1 phase in LoVo cells. There was no difference in p53 mRNA levels after Prx2 knockdown, but cycloheximide treatment showed an increased half-life of p53 in shPrx2 cell lines [67]. The authors discovered through IP and mass spectrometry that ribosomal protein RPL4 binds to Prx2. Ubiquitination assays were used to confirm RPL4 interaction with MDM2 and show that Prx2 increases ubiquitination of p53. Subcutaneous injection of control and shPrx2 cell lines resulted in higher tumor growth and the larger tumor volume in the control group. IHC analysis showed shPrx2 tumors had a higher expression of p53 [67]. Thus, Prx2 causes colorectal cancer growth in vitro, likely by facilitating degradation of p53.

Loss of Prx2 sensitizes CRC stem cells to chemotherapy [68]. IHC staining of 19 CRC patient tissues showed that Prx2 expression was significantly higher in CD133^+^/CD44^+^ tissues than in CD133^−^/CD44^−^ tissues. In spheroids of CD133^+^/CD44^+^ cells isolated from HCT116 and HT-29, the authors found higher expression of Prx2 compared to spheroids of CD133^−^/CD44^−^ cells. Western blot analysis showed significant downregulation of stemness-related proteins Oct4, Nanog, and Sox2 in CD133^+^/CD44^+^ cells isolated from HCT116 and HT-29 shPrx2 cell lines compared to those from control cell lines [68]. shPrx2 knockdown CD133^+^/CD44^+^ cells had lower migration and invasion in vitro compared to control stem cells. Orthotopic implantation of these two groups of cells in the cecal wall of nude mice resulted in significantly reduced liver metastasis in the shPrx2 group. The authors also found that shPrx2 CD133^+^/CD44^+^ cells had increased E-cadherin and decreased N-cadherin, Vimentin, Twist, and nuclear β-catenin than control CD133^+^/CD44^+^ cells [68]. Treatment of control or Prx2 knockdown CD133^+^/CD44^+^ cells with 500 μg/mL 5-fluororuracil or 100 μM oxaliplatin for 24 h showed increased cell death in the knockdown group as measured by annexin V flow cytometry analysis. DCFDA assay illustrated that chemotherapeutics induced significantly higher ROS in the Prx2 knockdown group. This was accompanied by increased DNA damage in shPrx2 CSC group cells than control CSCs as measured using an alkaline comet assay [68]. Similar experimental findings were reported by Wang et al. [69]. The authors sorted CD133^+^ and CD133^−^ cells from SW620, HT-29, and HCT116 and, using through Western blot analysis, found that Prx2 expression was higher in CD133^+^ cells in all three groups. Knockdown of Prx2 using shRNA reduced sphere formation of these cell lines by decreasing the mRNA and protein expression of CD44, CD133, and Nanog. Flow cytometry analysis showed that Prx2 depleted CD133^+^ cells were more prone to apoptosis by 5-FU [69]. CD133^+^ cells were isolated from control and HCT116 shPrx2 cell lines and injected into nude mice subcutaneously. Compared to the control group, the shPrx2 group had significantly smaller tumors at the endpoint of the study, suggesting the Prx2 contributes to tumorigenicity of colon cancer cells. Using Western blot, the authors found that, in HT-29 CD133^+^ cells, knockdown of Prx2 decreased and overexpression of Prx2 increased the expression of SMO and Gli1 proteins, suggesting Prx2 might regulate cancer stem cell properties via Hedgehog/Gli1 pathway [69]. 

Other studies have also shown that Prx2 depletion sensitizes CRC cells to ionizing radiation and chemotherapy [70,71,72,73]. Prx2 was silenced using siRNA in HCT116, Caco2, and T84 cell lines and clonogenic survival assay was performed after exposure to different doses of radiation [70]. Silencing Prx2 sensitized these cell lines to radiation. Oxaliplatin treatment before radiation was more effective in killing the shPrx2 cell line compared to control. HCT116, HCT116 shControl, and HCT116 shPrx2 cells were subcutaneously injected into flanks of nude mice, which were then irradiated with 2 Gy four days after inoculation. Six days post-radiation, the shPrx2 radiation group had significantly smaller tumors compared to day 1, and this effect was not seen in the other two groups [70]. Similar findings were reported by Xu et al. Annexin V staining showed increased apoptosis in HT-29 and HCT116 shPrx2 cell lines after 5-FU treatment [73]. Intraperitoneal injection of HCT116 control and shPrx2 cell lines into flanks of nude mice followed by no treatment or 5-FU treatment showed that the shPrx2 + 5-FU group had the longest survival. In addition, IHC staining of 49 patient specimens indicated that cyclophilin A (CypA) and Prx2 were upregulated in patients that did not respond to FOLFOX compared to patients that did respond [71]. The authors discovered through mass spectrometry that CypA interacts with Prx2. CypA overexpression decreased ROS levels in RKO cells, which could be partly rescued by knockdown of Prx2, indicating that Prx2 promotes reduction of CypA. Overexpression of CypA increased resistance of RKO cells to 5-FU and oxaliplatin, and this could be reversed by knockdown of Prx2 [71]. Similarly, overexpression of miR-200b-3p, which targets Prx2, sensitized LoVo cells to apoptosis by oxaliplatin, and this could be partly rescued by overexpression of Prx2 [72]. In a subcutaneous xenograft model, miR-200b-3p overexpression in LoVo cells inhibited tumor growth whereas silencing miR-200b-3p in SW480 promoted tumor growth. Implantation of extracted subcutaneous tumors into the cecum of nude mice showed that miR-200b-3p overexpression tumors developed significantly fewer metastatic nodules than control mice [72]. Thus, degradation of Prx2 reduces metastasis and enhances sensitivity of chemotherapeutics in CRC.

Loss of Prx2 also sensitized HCT116 cells to the antimalarial drug dihydroartemisinic (DHA) [74]. DHA treatment (15 μM) of HCT116 reduced Prx2 expression at mRNA and protein levels at 12 h and 24 h. DHA-induced ROS was confirmed by DCFDA staining in RKO and HCT116. DHA also induced ER stress-related proteins ATF4 and p-eIF2α in a time- and concentration-dependent manner. Prx2 knockdown further increased the sensitivity of HCT116 cells to DHA. Prx2 knockdown also enhanced the activation of JNK and p38 signaling pathways by DHA [74]. Combined treatment with oxaliplatin and DHA synergistically increased apoptosis in HCT116 and RKO cells. Furthermore, Prx2 was demonstrated to protect cells against DNA damage in checkpoint kinase 2 (CHEK2) null CRC [75]. The CHEK2 gene is involved in maintaining chromosomal stability and in homologous recombination repair. The authors treated CHEK2 null HCT116 cells with siRNA against Prx2 and found that combined loss of CHEK2 and Prx2 was lethal to the majority of HCT116 cells. Similarly, N-carbamoyl alanine (NCA, an inhibitor of Prx2) treatment of CHEK2 null HCT116 cells also reduced the viability of cells [75]. DCFDA assay showed increased ROS levels in HCT116 and CHEK2 null HCT116 cells with NCA treatment. However, NCA treatment caused more DNA damage in CHEK2 null HCT116 cells (as indicated by γ-H2AX staining) than in WT HCT116 cells. Accordingly, immunofluorescence staining showed a higher increase of cleaved caspase 3 in CHEK2 null HCT116 cells than WT HCT116 upon NCA treatment [75]. This suggests that Prx2 plays an important role in preventing oxidative stress-induced DNA damage in the absence of tumor suppressor CHEK2 in CRC. 

Prx2 promotes vasculogenic mimicry formation in CRC [76]. Vasculogenic mimicry refers to the vascular-like structures formed by cancer cells for blood supply independent of endothelial cells. IHC staining of Prx2 in 70 CRC patient tissues revealed that 70% of the tissues were positive for Prx2. Authors also performed double staining of CD34 and periodic acid–Schiff as markers for vasculogenic mimicry (VM). Pearson correlation analysis showed a positive correlation between Prx2 and VM formation [76]. Stable siPrx2 HCT116 cells were established, and recombinant VEGF was added to 3D culture of HCT116 cells to induce VM formation in vitro. siPrx2 cells had significantly fewer tubular structures than control cells. siPrx2 cells also had lower levels of p-VEGFR2 protein compared to control cells, suggesting Prx2 promotes VM formation by activating VEGFR2 [76]. Knockdown of Prx2 reduced the cell invasion of HCT116 caused by VEGF chemoattractant in Matrigel invasion assay. Thus, Prx2 supports the growth of aggressive tumors through VM formation.

**Figure 3:**
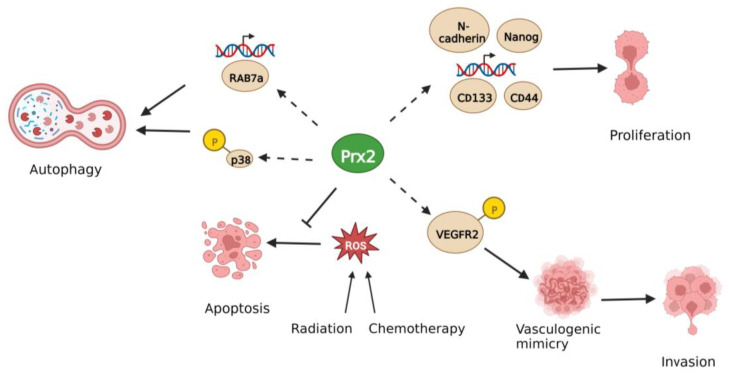
Prx2 promotes colorectal cancer progression. Prx2 increases autophagic flux, cancer stem cell expansion, vasculogenic mimicry, and inhibits ROS-induced apoptosis.

Prx2 loss inhibits autophagy in CRC [77]. Analysis of RNA-Seq data of HT-29 and SW480 control and siPrx2 cell lines followed by KEGG (Kyoto Encyclopedia of Genes and Genomes) analysis indicated enrichment of the FOXO pathway. Knockdown of Prx2 resulted in an increase of p21 and p27 proteins in Western blot analysis. shPrx2 cells had lower LC3B-GFP staining than non-targeting control cells [77]. Western blot analysis was used to show reduced LC3B II/LC3B I ratio and Beclin 1 along with increased Sqstm1/p62 in Prx2 knockdown cells compared to control. This indicates that Prx2 inhibits autophagosome formation. Western blot analysis also showed reduced p-p38 in shPrx2 cells. Treatment with 1 μM dehydrocorydaline chloride (DHC, a p38 MAPK activator) for 24 h rescued p-p38 to some extent in Prx2 cells. In addition, DHC also caused a decrease in p21 proteins to similar levels as non-targeting controls. Subcutaneous injection of control and shPrx2 cells into nude mice resulted in smaller tumor formation in the shPrx2 group [77]. Another study reported that oxiconazole (Oxi), an antifungal compound derived from imidazole, downregulates Prx2 in CRC cells to initiate autophagy and inhibit autolysosome formation by downregulating Rab7a [78]. When nude mice were subcutaneously injected with HCT116 cells followed by control or Oxi treatment (50 mg/kg/day), the Oxi treatment group developed significantly smaller tumors. Annexin V staining showed increased apoptosis in HCT116 and RKO after Oxi treatment. Oxi treatment increased cellular ROS levels as measured by active oxygen analysis kit. This increase in ROS and apoptosis could be inhibited by co-treatment with N-acetyl cysteine, suggesting that Oxi promotes ROS production to induce apoptosis [78]. Through immunofluorescence staining, the authors found that Oxi treatment increased autophagosome formation but not autolysosome formation in HCT116 and RKO cells. In Oxi-treated xenograft tissue, IHC showed stronger staining for LC3 than in the control group. Co-treatment with 3-mA (an autophagy inhibitor) rescued the decrease in cell viability caused by Oxi [78]. Oxi treatment also decreased Prx2 expression in a dose-dependent manner. Accordingly, Prx2 was found to be depleted in an Oxi-treated mouse xenograft model. Western blot analysis was used to examine lysosome–autophagosome fusion proteins and the authors found that Oxi decreased Rab7a expression. This could be partially reversed by Prx2 overexpression. Rab7a expression was also lower in the Oxi-treated mouse xenograft. Tandem monomeric mRFP-GFP tagged LC3 immunofluorescence assay in cells suggested that Oxi inhibits autolysosome formation through downregulation of Rab7a [78]. Thus, Prx2 regulates CRC progression through different signaling pathways, as summarized in Figure 3. 

#### 4.2.3. Prx3

Prx3 promotes survival and proliferation of lung adenocarcinoma cells in vitro. Prx3 was upregulated at mRNA and protein levels in 36 human lung adenocarcinoma (LUAD) samples compared to adjacent normal tissue [79]. Overexpression of tumor suppressor DACH1 resulted in downregulation of Prx3 transcript and protein in LUAD cell lines LTEP-α-2 and A549. DACH1-mediated downregulation of Prx3 resulted in reduced cell proliferation and anchorage-dependent colony formation in both cell lines. Similarly, downregulation of Prx3 increased susceptibility of NSCLC cells to radiation [80]. Knockdown of oncogene TP53-regulated inhibitor of apoptosis 1 (TRIAP1) in A549 and H460 cells sensitized these cells to irradiation. TRIAP1 knockdown cells had increased apoptosis and decreased cell invasion upon irradiation compared to wildtype cells. Irradiation of A549 and H460 increased transcript and protein levels of TRIAP1 and impaired the radiation-induced increase of antioxidants including Prx3, Prx4, and Prx6 [80]. Downregulation of Prx3 also increased the susceptibility of NSCLC cells to thiosemicarbazones. Myers group have reported that tridentate iron chelator triapine (Tp) (3-aminopyridine-2-carboxaldehyde thiosemicarbazone) oxidizes Prx3 in lung cancer lines (A549, H23, and H1703) and A2780 ovarian cancer cells [81]. Cytotoxicity of Tp correlated with Prx3 oxidation in the clonogenic survival of lung cancer lines. Knockdown of Prx3 further sensitized A549 cells to Tp [81].

Downregulation of Prx3 reduces viability of breast cancer cells in vitro. Knockdown of B7-H4 (also called VTCN1) decreased cell viability of MCF-7 and T47D cells [82]. This was associated with depletion of Prx3. Silencing Prx3 using siRNA caused increased intracellular ROS and decreased cell viability, similar to B7-H4 knockdown [82]. Thus, Prx3 likely protects breast cancer cells from oxidative stress-induced cell death.

Prx3 promotes stemness and survival of colon cancer cells [83]. qRT-PCR analysis showed increased Prx3 expression in CD133^+^ CSCs freshly isolated from eight patients with colon cancer compared to non-cancer stem cells. mRNA levels of Prx3 and CD133 in CSCs isolated from patient tissues showed significant positive correlation. Prx3 knockdown resulted in a decrease in the size of the CD133^+^ CSC population and sensitized the CSCs to 5-FU-induced cell death through mitochondrial dysfunction. Mice subcutaneously injected with CD133^+^ cells sorted from HT-29 shPrx3 showed reduced tumor volume and enhanced 5-FU-induced cell death compared with HT-29 shControl-injected mice [83]. Depletion of Prx3 resulted in a significant reduction in liver metastasis, colon metastasis, and local invasion in an orthotopic xenograft model produced by the injection of colon CSCs into the spleen and cecum of SCID mice. Chromatin immunoprecipitation assays showed that FOXM1 transcriptionally activates *CD133* and *Prx3* by binding to the promoter region of these genes. Overexpression of FOXM1 increased Prx3 and CD133 protein levels and expanded CD133^+^ population [83]. Thus, Prx3 supports CRC stem cells.

#### 4.2.4. Prx4

Prx4 increases proliferation and survival of prostate cancer cells. IHC staining of Prx4 in a human tissue microarray showed that Prx4 is upregulated in prostate adenocarcinoma compared to normal prostate [84]. Treatment of the LNCaP cell line with synthetic androgen R1881 led to a dose-dependent increased expression of Prx4 and PSA. In a LNCaP xenograft model, castration suppressed tumor growth. Western blot analysis of tumor lysates showed a significant downregulation of Prx4 and PSA in the castration group compared to control. Knockdown of Prx4 in LNCaP and DU145 cell lines reduced cell proliferation, migration, and invasion in vitro [84]. Phosphokinase array revealed that Prx4 depletion reduced activation of AKT and GSK3α/β signaling pathways. Prx4 depletion also sensitized LNCaP and DU145 cells to irradiation-induced cell death due to increased ROS accumulation (DCFDA assay) and DNA damage (indicated by γH2AX foci formation). Subcutaneous injection of control and Prx4 knockout DU145 cells resulted in smaller tumor volumes in the Prx4 knockout group. Similarly, irradiation of control and Prx4 knockout subcutaneous xenograft tumors suppressed tumor growth to a significantly higher extent in the Prx4 knockout group [84]. Thus, loss of Prx4 sensitizes prostate cancer cells to irradiation.

Prx4 is suggested to promote bone metastasis of prostate cancer and breast cancer [85]. Prx4 was depleted in MDA-MB-231 and PC3 cell lines, and conditioned media was collected to treat RAW264.7 cells. In vitro osteoclastogenesis assay showed suppression of osteoclast formation by knockdown of Prx4 compared to control cells, likely by reducing ERK phosphorylation and nuclear translocation of NFATc1. In an animal model, shPrx4 PC3 cells induced significantly smaller osteolytic lesions in tibia of CD-1 immunodeficient nude mice compared to control cells [85]. Little else is known about the mechanism of Prx4 secretion or the function of secreted Prx4 in metastasis of tumor cells.

Prx4 also promotes the progression of NSCLC. Knockdown of Prx4 decreases anchorage-independent colony formation and Matrigel invasion of A549 cells [45]. Phosphokinase array and Western blot analysis showed that loss of Prx4 represses c-Jun-mediated AP-1 activation. Subcutaneous injection and tail-vein injection of A549 cells into SCID mice resulted in smaller tumor volumes and fewer lung metastatic nodules in a Prx4 knockdown group compared to control [45]. Finally, Prx4 also promotes progression of CRC. IHC staining of 59 CRC patient samples showed that Prx4 is upregulated in CRC samples compared to adjacent normal samples, and Prx4 upregulation is positively correlated with infiltration depth, lymph node metastasis, and Dukes’ stage [86]. In 2-DE and mass spectrometry analysis of eight Stage 1 and eight Stage 4 CRC samples, significant upregulation of Prx4 in Stage 4 samples was confirmed [87]. Knockdown of Prx4 reduced cell proliferation and migration of DLD1 cells in vitro and subcutaneous tumor growth in vivo. Western blot analysis showed reduced phosphorylation of EGFR, RhoA, PKCα, and ERK in DLD1 shPrx4 cells compared to controls [87].

#### 4.2.5. Prx5

Prx5 promotes proliferation and EMT phenotype in gastric cancer cells [88]. Five-year survival data analyzed via the log-rank test indicated that overexpression of Prx5 was correlated with poor survival of gastric cancer patients. Expression of Prx5 significantly correlated with tumor size, lymph node invasion, and metastasis (TNM) stage [88]. Both proliferation and anchorage-dependent colony formation were higher in SNU-216 Prx5 overexpression cells than in parental SNU-216 cells. In Western blot analysis, E-cadherin was decreased, while Snail and Slug were increased in Prx5 overexpression SNU-216 cells [88].

Prx5 also promotes survival and EMT in NSCLC. Administration of non-thermal plasma therapy using plasma-activated medium (PAM) induced apoptosis in cancer cells by increasing ROS levels [89]. PAM was developed by treating A549 cell culture medium with low temperature plasma at 16.4 kV for 0, 60, 120, or 180 s. Knockdown of Prx5 enhanced ROS production, cytotoxicity, and inhibition of migration in A549 cells caused by PAM [90]. Western blot analysis showed that Prx5 knockdown in A549 reduced p-ERK and BCL2 and increased p-JNK and BAD proteins to promote apoptosis. Besides contributing to survival, Prx5 also promotes NSCLC growth and progression through its interaction with Nrf2 and Stat3. Prx5 could be pulled down using anti-Nrf2 antibody and vice versa in NSCLC and non-tumor lung tissues as well as in H1229 and A549 cell lysates [91]. Knockdown of Prx5 induced by H_2_O_2_ treatment decreased NQO1 protein levels in A549 and H1299 cells. Similarly, knockdown of Prx5 or NQO1 reversed the increase in cell proliferation of A549 and H1299 cells that was induced by H_2_O_2_ treatment. In analysis of patient samples, the authors found strong correlation of Prx5 mRNA with Nrf2 and NQO1 [91]. Subcutaneous injection of A549 cells into flanks of nude mice followed by no treatment or intra-tumoral injection of Nrf2 or Prx5 shRNA led to significantly reduced tumor growth in the shRNA treated group. Prx5 interaction with Stat3 was also reported by the Xue research group. qRT-PCR of 121 paired NSCLC tumor and adjacent normal samples revealed that 65% of the samples contained Prx5 promoter demethylation, and Prx5 promoter demethylation was associated with higher TNM stage [92]. Overexpression of Stat3 in H1299 cells pretreated with 100 µM H_2_O_2_ increased the Prx5 protein level, whereas knockdown of Stat3 decreased Prx5, suggesting that Stat3 is at least partially responsible for regulation of Prx5 expression. Overexpression of Prx5 in H1299 cells pretreated with 100 µM H_2_O_2_ increased in vitro migration and invasion. This was associated with a decrease in E-cadherin and increases in Vimentin, Nrf2, and NQO1, shown through Western blot analysis [92]. Thus, Prx5 plays a pro-tumorigenic role in NSCLC.

**Figure 4:**
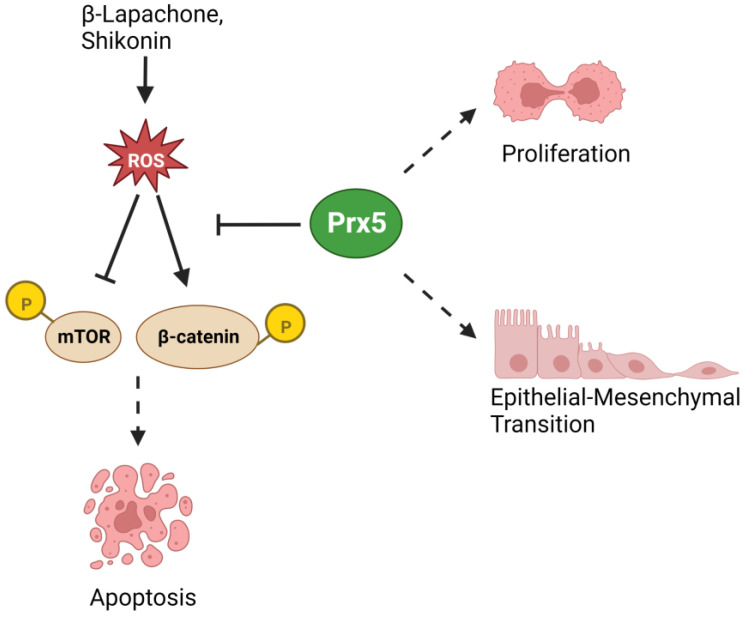
Prx5 promotes colorectal cancer progression. Prx5 suppresses proliferation, increases EMT, and inhibits ROS-induced cell death of CRC cells.

Prx5 promotes EMT phenotype in SW480 colon cancer cells [93]. Prx5 overexpression in SW480 cells increased cell proliferation, migration, and invasion rates in vitro. Western blot analysis showed that Prx5 overexpressing cells had lower E-cadherin and higher Vimentin, Slug, and Snail [93]. Knockdown of Prx5 using siRNA reversed the increase in cell proliferation, migration, invasion, and E-cadherin expression seen upon Prx5 overexpression. Prx5 also protects colon cancer cells from ROS-induced apoptosis [94]. β-lapachone, a compound extracted from the South American lapacho tree, is known to have anti-cancer activity. The authors performed bioinformatics analysis using the GEPIA website to show that Prx5 was upregulated in colon cancer compared to normal tissue. Increasing concentrations of β-lapachone treatment for 24 h showed SW480 shPrx5 cells to be more sensitive to this compound, whereas SW480 HisPrx5 cells were more resistant, compared to mock SW480 cells [94]. This was shown using a cell viability assay and annexin V staining. β-lapachone treatment decreased BCL2 (pro-apoptotic protein) expression in SW480 mock and shPrx5 cells. Dihydroethidium (DHE) staining indicated that β-lapachone treatment increased ROS levels in SW480 mock and shPrx5 cells [94]. In addition to ROS scavenging, Prx5 also regulates the Wnt/β-catenin pathway in response to β-lapachone. According to Western blot analysis, the ratio of p-Gsk3-β/Gsk3-β was higher and p-β-catenin/β-catenin was lower in Prx5-His cells than in mock and shPrx5 SW480 cells. The group also reported that Prx5 protects HCT116 and HT-29 cells from ROS-induced apoptosis [95]. Shikonin, a natural compound purified from the *Lithospermum erythrorhizon* plant, was previously reported to induce ROS in cancer cells. HCT116 and HT-29 cells were treated with increasing concentrations of Shikonin. JC-1 staining demonstrated that mitochondrial ROS increased in a dose-dependent manner. Similarly, DCFDA staining demonstrated a dose-dependent increase [95]. Using Western blot analysis, the authors found that Shikonin did not affect expression of proteins Prx2, Prx3, and Prx6, but Prx1 was increased and Prx5 was decreased with higher concentrations of Shikonin. Increasing concentrations of Shikonin resulted in a dose-dependent decrease of p-mTOR/mTOR ratio in HT-29 cells [95]. In an MTT assay, HisPrx5 HT-29 cells had higher viability than control mock HT-29 cells with increasing Shikonin concentrations. This increased resistance to cell death was also confirmed by DHE staining and annexin V flow cytometry analysis. Finally, HisPrx5 overexpression prevented a decrease in p-mTOR/mTOR ratio upon Shikonin (Figure 4) [95]. Thus, Prx5 plays a potential role in suppressing apoptosis in colon cancer.

#### 4.2.6. Prx6

Prx6 promotes NSCLC growth and survival. Overexpression of Prx6 increased cell proliferation, invasion, and migration of A549 cells; these were decreased by knockdown of Prx6 [96]. Western blot analysis indicated that Prx6 overexpression promotes EMT by downregulating E-cadherin and upregulating Vimentin, Twist, β-catenin, and c-Myc. In a subcutaneous A549 xenograft model, Prx6 overexpression increased tumor growth while Prx6 knockdown suppressed tumor growth [96]. A positive correlation between CD133 and Prx6 protein expression in NSCLC patient samples is reported [97]. Knockdown of Prx6 decreased the CD133^+^/ABCG2^+^ population in H1299 and A549 cells as well as the sphere formation ability of these cancer stem-like cells. Knockdown of Prx6 also reduced the IC_50_ of cisplatin for H1299 and A549 CSCs by 50% [97]. Another study also reported a positive association between Prx6 and drug resistance. Two-dimensional gel electrophoresis of six pairs of pretreatment fresh primary lung adenocarcinoma tumors with varied chemotherapy responses revealed that Prx6 was upregulated in chemo-resistant tumors [98]. Furthermore, Prx6 promotes growth of NSCLC. Withangulatin A (WA) is a small molecule isolated from *Physalis angulata* var. *villosa* and is reported to reduce proliferation of cancer cells. Stable isotope labeling by amino acids in cell culture and activity-based protein profiling in H1975 cells identified Prx6 as a direct target of WA [99]. WA covalently binds to Prx6 to inhibit its function and increases the production of ROS as indicated by DCFDA assay in H1975 cells. Subcutaneous injection of wildtype and Prx6 knockout (Prx6 KO) H1975 cells resulted in significantly lower tumor volume in the Prx6 KO group. WA treatment had no significant effect on proliferation, GPx activity, and PLA_2_ activity in H1975 Prx6 KO cells in vitro, or in growth of Prx6 KO tumors in vivo, confirming that WA acts through Prx6 [99]. 

Prx6 also contributes to the progression of CRC. IHC staining of Prx6 in a CRC patient tissue microarray showed that Prx6 was upregulated in node-positive CRC compared to node-negative CRC [100]. Stable knockdown of Prx6 reduced cell migration and invasion in HCT116 cells. Prx6 knockdown also caused downregulation of N-cadherin, CDK1, and Twist1 in HCT116. This was associated with reduced phosphorylation of PI3K, AKT, p38, and p50, as indicated by Western blot analysis [100]. Treatment of HCT116 with NAC, PI3K/AKT inhibitor wortmannin, and p38 MAPK inhibitor SB203580 resulted in a decreased trimethylation of histone H3 lysine 4 (H3K4me3) of Prx6 promoter [100]. This suggests a role for PI3K/AKT pathway in upregulation of Prx6 in CRC.

## 5. Conclusions and Perspectives

In summary, Peroxiredoxins play a primarily pro-tumorigenic role in human cancers (Table 2). Prxs not only promote the survival of cancer cells and cancer stem cells through ROS scavenging in tumors, which are an environment with high oxidative stress, but they also protect tumor cells against additional stress induced by ionizing radiation and chemotherapeutics. In addition, Prxs promote proliferation, migration, and invasion of cancer cells through modulation of a number of signaling pathways, including Wnt/β-catenin, JNK, and AKT. Furthermore, Prxs inhibit tumor suppressors, such as p53. Prx1 and Prx2 are also known to promote angiogenesis and vasculogenic mimicry in CRC.

Development of specific inhibitors of Prxs is proving challenging since Prxs are one of many proteins that contain active-site thiols. Therefore, continued investment and research into downregulation of specific Prxs through additional strategies, such as gene therapy, is critical to translate this potential for improved cancer prevention and treatment into reality. Similarly, exciting findings await regarding the role of Prxs in regulating tumor-promoting inflammation and immune evasion. As observed with Prx1 in breast cancer, it will not be surprising to find opposing roles of Prxs in tumor cells compared to stromal cells. Finally, detailed preclinical studies should be undertaken to elucidate the mechanistic roles of Prxs in remodeling tumor microenvironments. For all these processes, the roles of intracellular as well as extracellular Prx must be delineated. Once specific inhibitors for expression or activity of Prxs are identified, the next step would be to explore their additive and synergistic effects in combination with clinical drugs. Understanding these phenomena will provide the scientific and pharmaceutical communities additional knowledge and tools to tackle cancers. 

## Figures and Tables

**Table 1:** Summary of conservation of Prx homologues in vertebrate model organisms relative to human Prx (generated using HomoloGene).

	Percent Sequence Similarity to Human Prxs											
	Prx1		Prx2		Prx3		Prx4		Prx5		Prx6	
Species	DNA	Protein	DNA	Protein	DNA	Protein	DNA	Protein	DNA	Protein	DNA	Protein
*Macaca mulatta*	98.5	99.5	98.1	100	97.5	97.7	98.4	98.5	97.8	99.2	98.4	98.7
*Mus musculus*	90.8	95.5	88.2	93.4	84.2	86.3	89.1	95	86.1	87.2	87.4	89.7
*Rattus norvegicus*	91.3	97.5	87	93.4	83.2	85.2	90.3	94.5	85.3	88.1	88.8	91.5
*Danio rerio*	73.9	81.3	72.8	76.6	66.8	75.4	74.8	88.7	64	61.3	67.3	73.6

**Table 2:** Prxs in carcinogenesis and cancer progression.

Prxs	Cancers	Pro- or Anti-tumor	Mechanism/Pathway	References
Prx1	Lung	Pro-tumor	Downregulation of E-cadherin	[44]
		Pro-tumor	Protection against oxidative stress-induced cell death and inhibition of ASK1-JNK	[46,47]
	Prostate	Pro-tumor	Protection against oxidative stress-induced cell death and interaction with TPD52	[50]
		Pro-tumor	Activation of AR signaling	[51]
	Colorectal	Pro-tumor	Cullin-5 neddylation-mediated NOXA degradation	[54]
		Pro-tumor	Increased inflammation	[36]
	Breast	Pro-tumor	Prevention of Rad51 oxidation to promote homologous recombination	[58]
		Anti-tumor	Mediation of TAGLN2 activity	[60]
		Anti-tumor	Maintenance of redox homeostasis	[37]
		Anti-tumor	Inhibition of LOX secretion and extracellular matrix remodeling	[59]
		Anti-tumor	Inhibition of pro-tumorigenic macrophage differentiation	[61]
		Anti-tumor	Inhibition of pro-tumorigenic fibroblast differentiation	[38]
		Anti-tumor	Increased survival of Natural killer cells	[62]
Prx2	Lung	Pro-tumor	Upregulation of Vimentin, Slug and, activation of AKT/mTOR	[63,64]
		Pro-tumor	Activation of Hedgehog, Notch, Wnt/β-catenin	[65]
		Pro-tumor	Maintenance of SIRT1 activity through inhibition of AMPK	[66]
	Colorectal	Pro-tumor	Degradation of p53	[67]
		Pro-tumor	Increased stemness, radioresistance and chemoresistance	[68,69,70,71,72,73]
		Pro-tumor	Stabilization of β-catenin in APC mutant cells	[39]
		Pro-tumor	Protection against oxidative stress-induced cell death	[74,75]
		Pro-tumor	Formation of vasculogenic mimicry	[76]
		Pro-tumor	Inhibition of autophagy	[77,78]
Prx3	Lung	Pro-tumor	Radioresistance and chemoresistance	[80,81]
	Breast	Pro-tumor	Protection against oxidative stress-induced cell death	[82]
	Colorectal	Pro-tumor	Increased stemness and chemoresistance	[83]
Prx4	Prostate	Pro-tumor	AR activation and radioresistance	[84]
	Prostate, Breast	Pro-tumor	Activation of ERK/NFATc1	[85]
	Lung	Pro-tumor	Activation of c-jun/AP-1	[45]
		Pro-tumor	Protection against oxidative stress-induced cell death and increased activation of NF-κB and AP-1 signaling	[40]
		Pro-tumor	Increased cell transformation, proliferation and increase in intra-tumoral M2 macrophage infiltration	[41]
	Colorectal	Pro-tumor	Activation of EGFR, RhoA, PKCα, ERK	[87]
		Pro-tumor	Protection against oxidative stress-induced cell death and increase in intra-tumoral immune infiltration	[42]
Prx5	Lung	Pro-tumor	Protection against oxidative stress-induced cell death	[90]
		Pro-tumor	Upregulation of Vimentin, Nrf2, NQO1	[91,92]
	Colorectal	Pro-tumor	Upregulation of Vimentin, Slug	[93]
		Pro-tumor	Protection against oxidative stress-induced cell death	[94,95]
Prx6	Lung	Pro-tumor	Upregulation of Vimentin, Twist, β-catenin	[96]
		Pro-tumor	Protection against oxidative stress-induced cell death	[99]
		Pro-tumor	Increased stemness and chemoresistance	[97,98]
		Pro-tumor	Increased PLA2 activity	[43]
	Colorectal	Pro-tumor	Activation of PI3K/AKT and upregulation of N-cadherin	[100]

## Data Availability

Not applicable.

## Abbreviations

2-DE	two-dimensional gel electrophoresis
5-FU	5-fluorouracil
AOM	azoxymethane
AR	androgen receptor
CRC	colorectal cancer
CSC	cancer stem cell
DCFDA	2′,7′-dichlorodihydrofluorescein diacetate
DMBA	7,12-dimethylbenz[a]anthracene
DSS	dextran sulfate sodium
EMT	epithelial-mesenchymal transition
GPx	glutathione peroxidase
GSH	glutathione
GST	glutathione-s-transferase
IHC	immunohistochemistry
IP	immunoprecipitation
LOX	lysyl oxidase
LUAD	lung adenocarcinoma
NAC	N-acetyl cysteine
NSCLC	non-small cell lung cancer
PAM	plasma-activated medium
PLA_2_	phospholipase A_2_
Prx	peroxiredoxin
PSA	prostate-specific antigen
qRT-PCR	quantitative real-time polymerase chain reaction
ROS	reactive oxygen species
Srx	sulfiredoxin
Trx	thioredoxin
UC	ulcerative colitis
WA	withangulatin A
